# Revisiting Non-operative Treatment of Lateral Compression Pelvic Fractures, Analysis of Rehabilitation, and Radiologic Outcomes in a Historical Cohort Using Today’s Association of Osteosynthesis (AO) Stability Criteria

**DOI:** 10.7759/cureus.32101

**Published:** 2022-12-01

**Authors:** Claudio Rojas, Ernesto Ewertz, Jose Hormazabal

**Affiliations:** 1 Orthopaedics and Traumatology, Hospital del Trabajador, Santiago, CHL; 2 Orthopaedics and Traumatology, Clinica Davila, Santiago, CHL

**Keywords:** ao-ota classification, non operative, lateral compression injury, orthopedic treatment, pelvic fracture

## Abstract

Background: Type I lateral compression pelvic fractures (LC-I) have historically been treated conservatively. Inferior outcomes in a distinctive subset of these injuries have been reported, therefore their management has shifted towards surgery. Revisiting the historical series of LC-I allows us to determine whether non-operative management of these unstable patterns results in poorer outcomes. The objective was to evaluate the differences in the rehabilitation progress, fracture consolidation, and displacement in non-operatively treated LC-I fractures that would be considered unstable using today's Association of Osteosynthesis (AO) criteria.

Methods: We conducted a retrospective review of conservatively treated LC-I injuries in a single-level I trauma center between June 2010 and June 2014. Patients were distributed in stable (group A) and unstable (group B) groups according to the 2018 AO classification. Time to walk independently (TWI), time to return to work (TRW), fracture consolidation, and displacement were analyzed.

Results: 34 patients, mean age of 45.5 ±14.5 years, were included. Mean TWI in groups A and B were 71.2 ±31.9 and 105.9 ±50.9 days (p=0.027). Mean TRW was 106 ±51.3 and 157 ±84 days in groups A and B, respectively (p=0.038). A difference in mean TWI and TRW of 34.7 and 51.3 days between groups was observed. No significant differences in fracture consolidation or displacement were observed.

Conclusion: Unstable fractures presented significantly longer TWI and TRW. The revised AO classification contributes to the identification of fracture patterns that correlate with prolonged rehabilitation in which additional treatment strategies might be considered.

## Introduction

Lateral compression (LC) fractures are among the most common pelvic ring injuries, representing between 57% to 63% of these injuries [1,2]. The initial studies of Tile et al. grouped these fractures according to their primary mechanism of injury and direction of instability [3]. These injuries represent a spectrum of continuous damage to pelvic structures ranging from stable to rotationally and vertically unstable fractures [4,5]. Type I lateral compression fractures (LC-I) have historically been managed nonoperatively due to the theoretic integrity of ligaments that provide structural stability [6,7].

In the last decade, this assertion has been questioned. Bruce et al., in a series of 117 patients, reported fracture displacement in as many as 40% of LC-I injuries with complete posterior sacral disruption [8]. Sagi et al. reported that 35% of Orthopaedic Trauma Association (OTA) B2 fractures stressed under anesthesia were unstable enough to require operative management [9]. Tosoudinis et al. reported significant improvements in length of hospital stay, time to independent pain-free mobilization, post-manipulation pain levels, and opioid requirements in surgically treated LC-I patients [10]. In light of this evidence, a revised pelvic fracture classification from the Association of Osteosynthesis (AO) group was recently published, suggesting surgical management according to the severity of the posterior arch injury. [11].

Currently, the evaluation of how non-operatively treated patients with unstable fractures perform is difficult due to a more aggressive approach toward surgery. Revisiting the historical series of conservatively treated LC-I fractures allows us to determine whether the re-defined unstable patterns present poorer outcomes.

The objective of this study is to evaluate the differences in the rehabilitation progress, fracture consolidation, and displacement in non-operatively treated LC-I fractures that would be considered unstable per today's AO stability criteria.

This article was previously posted to the Research Square preprint server on November 1, 2021.

## Materials and methods

Institutional board review approval was obtained and the requirement for written informed consent was waived in relation to this study. We conducted a retrospective cohort study of a historical series of non-operatively treated LC-I patients between June 2010 and June 2014 in a single-level I trauma-workers insurance center.

We revised all patients admitted with pelvic fracture diagnoses. Conservatively treated LC-I fractures were included. Exclusion criteria were defined as the presence of associated injuries that interfered with immediate standing and walking (severe traumatic brain injuries, upper extremity injuries that precluded walking aids, lower extremity injuries that required unloading) and incomplete radiological studies in the digital imaging software (Xero Viewer 8.1.2 system, Agfa Healthcare, Mortsel, Belgium). Three independent investigators including the senior author analyzed the radiological images.

Fractures were grouped according to the 2018 AO classification system into stable fractures (61 B1.1) in group A and unstable fractures (61 B2.1) in group B [12].

Patients were followed-up in the office by trained pelvic trauma surgeons. The rehabilitation progress was assessed by analyzing the time to walk independently (TWI) without walking aids and the time to return to work (TRW) extracted from digital clinical chart registries. Consolidation of posterior sacral fracture and displacement (>10 mm) in any direction were determined by comparing pelvic AP inlet and outlet views and CT scans at admission and six months follow-up, and analyzed as a binary variable.

Statistical analysis was performed using Stata Statistical Software (Stata Corp, College Station, TX, USA). The normal distribution of the population was evaluated using the Shapiro-Wilk test. Differences between groups were evaluated using Student's t-test for continuous and Fisher's exact test for categorical variables. A p-value for statistical significance was set at <0.05. Age, TWI, and TWR values are presented in means and standard deviation (SD).

## Results

Between June 2010 and June 2014, a total of 116 patients with pelvic ring fractures were admitted to our institution. Sixty-six patients met the inclusion criteria. Twenty-seven patients presented associated injuries that precluded immediate weight bearing, and in five patients the use of walking aids was not registered. Thirty-four patients were selected for the final analysis.

Seventeen women and 17 male patients with a mean age of 45.5 ± 14.5 years were included. Fifteen patients were considered stable and assigned to group A and 19 patients were considered unstable and assigned to group B. Patients in group A and group B presented similar age (49.2 ± 16.09 years vs 43 ± 12,8 p = 0.89) and gender distribution (M:F=7:8 vs M:F=11:8 ; p=0.7)

A complete description of anterior and posterior arch fracture characteristics is presented in Table 1. In group A, 14 patients presented unilateral pubic rami fractures; eight of them were multifragmentary. No comminuted posterior arch fractures were observed in this group. In group B, 12 patients presented unilateral rami fractures; three of them were contralateral to the posterior pelvic injury and seven had bilateral pubic rami fractures. Multifragmentary anterior arch fractures were observed in seven patients. In posterior arch injuries, 14 simple and five multifragmentary sacral fractures were observed.

**Table 1:** Radiologic characteristics and 2018 AO classification AO: Association of Osteosynthesis

		Group A	Group B
Number of patients		15	19
Anterior arch injury characteristics			
	Simple unilateral	6	6
	Simple bilateral	0	4
	Multifragmentary unilateral	8	6
	Multifragmentary Bilateral	1	3
Posterior arch injury characteristics			
	Incomplete fracture	15	0
	Complete simple fracture	0	14
	Complete multifragmentary fracture	0	15
AO 2018 classification			
	61B1.1	15	0
	61B2.1	0	19
Qualifications			
	a Ipsi or unilateral pubic rami fractures	12	9
	b Bilateral pubic rami fractures	0	7
	c Contralateral pubic rami fractures	0	3
	e Parasymphyseal fracture	3	0

Three patients presented delayed consolidation and one patient presented significant displacement at the six-month follow-up, all belonging to the unstable fracture group (group B). No significant differences in consolidation or displacement were observed between groups. Detailed clinical and radiological values are presented in Table 2.

**Table 2:** Clinical and radiological variables TWI: Time to walk independently, TRW: Time to return to work † Values: mean, range , ‡ Values: number of patients * statistically significant

	Group A	Group B	p-value
Clinical evolution †			
TWI (days)	71,2 (53-88)	105,94 (81-130)	0.027*
TRW (days)	106,2 (83-129)	157,5 (109-160)	0.038*
Radiological evolution ‡			
No displacement	15	18	0.7
Displacement	0	1	
Consolidation	15	16	0.25
Non-union	0	3	

The mean TWI in groups A and B was 71.2 ± 31.9 and 105.9 ± 50.9 days. respectively (p=0.027). The mean TRW 106 ± 51.3 vs 157 ± 84 days (p=0.038), respectively. Group A presented a decrease in mean TWI of 34.7 days and in mean TRW of 51.3 days when compared with group B (as seen above in Table 2).

## Discussion

Despite significant advances in trauma surgery in the last few decades, the understanding of biomechanics and the prediction of pelvic fracture stability remains controversial [8,12]. Even though the Young & Burgess classification aids in comprehending the injury mechanisms and anticipating associated lesions, it is insufficient to accurately predict how these fractures behave under physiological loads. Previously, the AO based their classification on the direction in which instability of the pelvic ring was presumed; B2.1 and B2.2 fractures were considered rotationally unstable with maintained vertical stability; albeit also failing to accurately predict future displacement [13]. Given the evidence available following the initial description of Pennal et al. and through the first decade of the 21st century, LC-I fractures used to be managed nonoperatively [14]. However, new evidence gave rise to the question of whether some of them would benefit from surgical fixation.

The wide spectrum of fracture severity, recoil phenomena, displacement, and differences in clinical outcomes makes these a particularly difficult group of fractures to evaluate [8,9,15-17]. On the other hand, the prediction of the dynamic behavior of these injuries with current static imaging methods, adds to the complexity of decision-making. Dynamic stress testing under anesthesia is useful but impractical to apply in all patients.

Displacement has been associated with poorer outcomes in pelvic fractures and the time needed to progress in physical rehabilitation has important physical and psychological consequences for these patients [18-22]. In 2018, the AO classification system incorporated the differentiation of stable and unstable lateral compression fractures primarily based on the severity of the posterior sacral fracture [11].

In this study we evaluated the rehabilitation milestones of TWI and TRW; consolidation and fracture displacement of non-operatively treated LC fractures which under today's scope would be considered unstable.

An objective assessment of displacement in pelvic fractures is difficult. The complex three-dimensional anatomy and the impossibility of getting standardized comparable imaging in the trauma setting make the evaluation unreliable [23]. In this study, we conducted a subjective radiologic analysis relying on the ability of trained pelvic trauma surgeons to identify fracture displacement and consolidation. Only the unstable fracture patterns (group B) presented fracture displacement and delays in consolidation, though no significant differences between groups were observed. On the other hand, stable and unstable fractures presented significant differences in TWI and TRW. This suggests that the 2018 AO criteria help identify patients in whom a nonoperative approach results in prolonged rehabilitation periods, though whether the surgical treatment of these unstable fractures improves outcomes remains unclear [19,24-29].

This study has many limitations. The retrospective nature of the study doesn't allow for functional scores or patient-reported outcomes measure scores to be applied. The sample is small and composed entirely of workers entitled to compensation benefits which limit the external validation of the results.

## Conclusions

This study adds to the growing evidence demonstrating variable outcomes in the highly heterogeneous LC-I fracture group. The revised 2018 AO classification system contributes to the identification of unstable fracture patterns that correlate with longer rehabilitation and work absentee times in which additional treatment strategies might be considered. Further studies should be conducted to determine the functional impact of surgery on these patients.
